# Profile of Maternal Mortality of COVID-19 in Ceará, in the Period of 2020

**DOI:** 10.3390/ijerph20054645

**Published:** 2023-03-06

**Authors:** Sheyla Martins Alves Francelino, Italla Maria Pinheiro Bezerra, Sabrina Alaide Amorim Alves, Francisco Naildo Cardoso Leitão, Mauro José de Deus Morais, José Lucas Souza Ramos, João Batista Francalino da Rocha, Thaiany Pedrozo Campos Antunes, Fabiana Rosa Neves Smiderle, Luíz Carlos de Abreu

**Affiliations:** 1 Programa de Pós-Graduação em Ciências da Saúde, Centro Universitário FMABC (FMABC), Santo André 09060-870, Brazil; 2 Curso de Enfermagem e Laboratório de Escrita Científica, Escola Superior de Ciências da Santa Casa de Misericórdia de Vitória (EMESCAM), Vitória 29027-502, Brazil; 3 Programa de Pós-Graduação em Políticas Públicas e Desenvolvimento Local, Escola Superior de Ciências da Santa Casa de Misericórdia de Vitória (EMESCAM), Vitória 29027-502, Brazil; 4 Programa de Pós-Graduação em Cuidados Clínico em Enfermagem e Saúde, Universidade Estadual do Ceará (UECE), Fortaleza 60714-903, Brazil; 5 Laboratório Multidisciplinar de Estudos e Escrita Científica em Ciências da Saúde (LaMEECCS), Universidade Federal do Acre (UFAC), Rio Branco 69920-900, Brazil; 6 Programa de Pós-Graduação em Ciências Médicas, Faculdade Medicina Universidade de São Paulo, São Paulo 01246-903, Brazil; 7 School of Medicine, University of Limerick, V94 T9PX Limerick, Ireland

**Keywords:** COVID-19, pandemic, maternal death, pregnancy, postpartum period

## Abstract

The aim of this paper is to describe the profile of maternal mortality of COVID-19 in the state of Ceará, Brazil, in the period 2020. Ecological, exploratory, cross-sectional study, with secondary data from the Influenza Epidemiological Surveillance Information System, were made available by the Obstetric Observatory Brazilian COVID-19. A total of 485 pregnant and postpartum women were included, and the analysis considered the notifications from the year 2020. The variables of interest and the outcome (death/cure by COVID-19) were analyzed in a descriptive way. Most pregnant and postpartum women were between 20 and 35 years old, brown/white skin color and residing in an urban area. The proportion of deaths was 5.8% in the year 2020. In that period, the rates of hospitalization in the ward increased by 95.5%, 12.6% of hospitalization in the Unit of Intensive Care (ICU), and 7.2% needed invasive ventilatory support. Maternal mortality from COVID-19 suggests an emergency in terms of the development of health actions and policies due to the aggravation and risks due to this disease.

## 1. Introduction

COVID-19 has devastating impacts worldwide, causing deaths from this disease, generating disparities, economic and social, mainly for the more vulnerable population [1]. Long-term research indicates that in Brazil, during the 44 epidemiological weeks of 2020, the country accounted for 7714.819 cases and 195,742 deaths, becoming the epicenter of the pandemic [2,3].

Among the risk groups, pregnant and postpartum women are highlighted, as their physiological changes increase the risk of infections for them [4]. A study on the occurrence of COVID-19 in the prenatal period showed the occurrence of cases from asymptomatic to severe clinical conditions, including typical pathways or even nonspecific manifestations, with systemic and gastrointestinal symptoms [5]. However, at the beginning of the pandemic, research did not define pregnant women as a risk group for complications and deaths. This fact is probably due to the low occurrence of COVID-19 in pregnant women [6].

A study carried out by the Group of Brazilian Studies on COVID-19 and Pregnancy, through data analyzed by the Influenza Epidemiological Surveillance System (in Portuguese, SIVEP Gripe) found that 978 pregnant and postpartum women were diagnosed with Severe Acute Respiratory Syndrome (SARS) due to COVID-19 and of these, 124 died (fatality rate of 12.7%). The data showed an association of deaths in this population with comorbidities, such as obesity, diabetes, and cardiovascular diseases, highlighting serious operational failure regarding the care of pregnant women, 5% of the women did not receive any type of ventilatory assistance, 28% did not have access to an intensive care unit (ICU) bed, and 36% were not intubated or mechanically ventilated [7].

The COVID-19 pandemic increased barriers to accessing prenatal and postpartum maternity care, especially for poor and black women, raising concerns about serious maternal mortality during the pandemic [8].

In this context, this study is justified by the substantial increase in maternal mortality during the pandemic period, therefore, investigating the areas of risk and exploring the reality are needed, highlighting the need for intervention in a population in which maternal mortality is mostly preventable. 

It seems that the COVID-19 context is changing the maternal mortality profile and effort to improve the health of these women is needed. Knowing this profile is the base to develop health practices and policies to guide health promotion. Thus, we aimed to describe the profile of maternal mortality from COVID-19 in the State of Ceará, Brazil, in the period 2020.

## 2. Materials and Methods (Track Changes) 

### 2.1. Design

This is an ecological, exploratory, cross-sectional study with exploratory analysis of data on maternal mortality from COVID-19 in Ceará, Brazil.

### 2.2. Place, Period, and Population of the Study

The scenario analyzed was the state of Ceará, located in the Northeast Region, Brazil. Data from detected cases of COVID-19 in pregnant and postpartum women who died in the period from 1 April to 31 December, 2020, were used. Such periods are justified by the disclosure of cases of notification of the epidemiological weeks in the information systems in health.

Data regarding deaths from COVID-19 in pregnant and postpartum women in the state of Ceará were extracted from the Influenza Epidemiological Surveillance Information System (SIVES-Gripe), released by the Brazilian Obstetric Observatory COVID-19 (OOBr COVID-19) [9]. The OOBr COVID-19 corresponds to a panel with case analyses of pregnant and puerperal women notified in the SIVEP-Gripe [10].

The study population comprised all cases of deaths from COVID-19 in pregnant and puerperal women notified in the Influenza Epidemiological Surveillance Information System (SIVES-Flu), in the period of 2020. It included females with age equal to or greater than ten years and less than 50 years, residing in the state of Ceará, and their death occurred in the period of 2020.

The Maternal Mortality Ratio was calculated in the state of Ceará, in the period of 2020, with data available in the virtual environment of the Department of Health of the SUS (DATASUS). This calculation considers the number of deaths of resident women due to causes and conditions considered to be maternal death (direct and indirect), divided by the number of live births to resident mothers and after, multiplied by 100,000 [11].

### 2.3. Data Collection Instruments and Variables

To guide data collection, a spreadsheet containing the following variables was used: sociodemographic profile—age, race/color, education, area of residence. Epidemiological profile—gestational age, comorbidities (cardiovascular, diabetes, neuropathies, lung disease, kidney disease, obesity). Care profile—confirmed cases, confirmed cures, confirmed deaths, ward admission, ICU admission, non-invasive ventilatory support, invasive ventilatory support.

A semi-structured form recording the geographic unit of the federation with coding adopted by the Brazilian Institute of Geography and Statistics (IBGE) was also used as a data instrument to identify Brazilian regions, states, and municipalities in order to obtain information that meets the objectives of this study.

### 2.4. Data Analysis

The data were stored in an electronic bank created in the Microsoft^®^ Excel program. Then, a simple descriptive analysis was performed in absolute numbers and percentages.

### 2.5. Ethical Aspects

The research was carried out with secondary data from the Brazilian Obstetric Observatory COVID-19, without identifying the participants, in compliance with the ethical aspects of Resolutions 466/2012 and 510/2016 of the National Health Council (CNS), with a waiver of submission to the Ethic.

## 3. Results

Table 1 shows the evolution of COVID-19 cases in pregnant and postpartum women in the state of Ceará in the period 2020. In total, 485 confirmed cases of COVID-19 were reported in pregnant and postpartum women in this period, and 84.7% of the confirmed cases progressed to cure. Regarding the number of confirmed deaths in pregnant and postpartum women due to COVID-19, the percentage was 5.8%. The study reveals high rates of hospitalization, with 95.5% in the ward, 12.6% in the Intensive Care Unit (ICU), and 7.2% of pregnant and postpartum women requiring invasive ventilatory support.

Table 2 shows the sociodemographic characteristics, highlighting that ages ranged between 20 and 35 years old, configuring a group of young-adult pregnant and postpartum women. There was a predominance of pregnant and postpartum women living in urban areas, 71.1%, compared to 14.8% in rural areas. As for race, 70.3% self-declared to be mixed race, 0.35% white, and 0.4% black. Among the participants, 16.9% had completed high school, and 6% had completed middle school.

Table 3 presents data on the epidemiological profile of pregnant and postpartum women due to COVID-19 in the state of Ceará in the period 2020, with most women in the 3rd gestational trimester 66.0%, 9.3% in the 2nd gestational trimester, and 16.9% were postpartum women.

Regarding the variables related to comorbidities shown in Table 3, there was a predominance of pregnant and postpartum women affected by kidney disease, 13.8%, 4.3% for cardiovascular diseases, and 3.7% of them were diabetic. Of these, 0.2% had comorbidities related to neuropathies, and 1.0% were obese.

Regarding the data on the mortality rate of maternal death, we considered the death of women during pregnancy and/or up to 42 days after the end of pregnancy. Thus, it was 386.6 maternal deaths per 100,000 live births in the state of Ceará in 2020.

**Figure 1:**
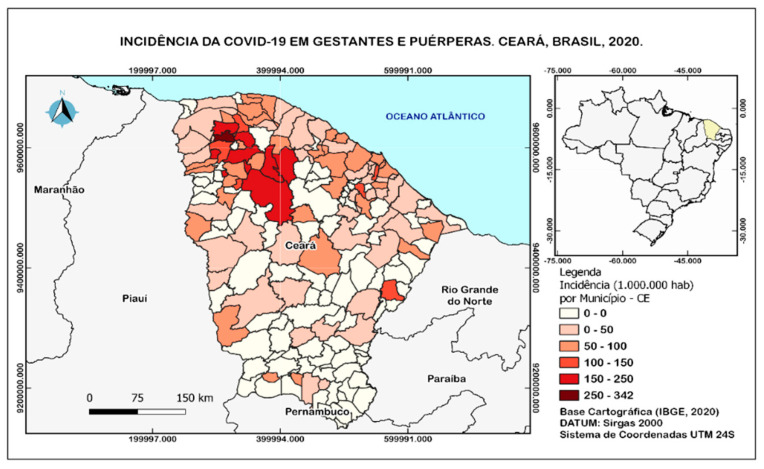
Incidence of COVID−19 in pregnant and postpartum women in the state of Ceará, Brazil, 2020. Label: Incidence (10,000 inhabitants) per municipality−CE. Cartographic base (IBGE, 2020). DATUM: Sirgas 2000, 24S UTM coordinate system.

There was a higher concentration of COVID-19 incidence rates in pregnant and postpartum women in the macro-region of Sobral, with a concentration in the cities of Santa Quitéria, Irauçuba, Sobral, Massapé, Groairas, Senador Sá, and Uruoca, as shown in Figure 1. We can observe that new cases of COVID-19 in pregnant and postpartum women were maintained.

**Figure 2:**
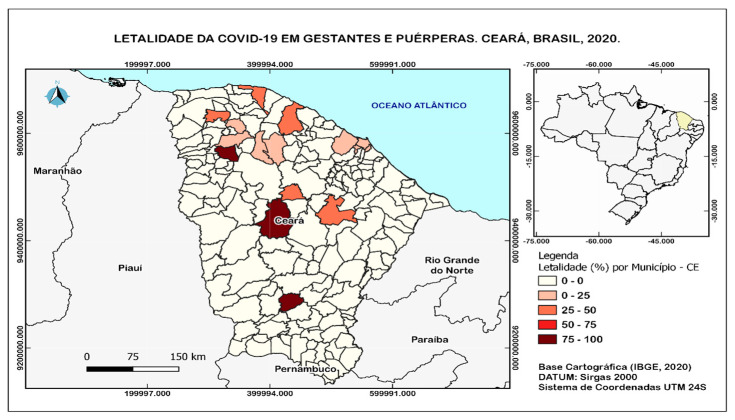
Lethality of COVID−19 in pregnant and postpartum women in the state of Ceará, Brazil, 2020. Label: Lethality (%) per municipality−CE. Cartographic base (IBGE, 2020). DATUM: Sirgas 2000, 24S UTM coordinate system.

There was a cluster of lethality for COVID-19 in pregnant and postpartum women in the region of the wilderness of Sobral, being Cariré, wilderness of Canindé, and the center-South region of Cariri, in the city of Jucas (75—100 per 10,000 inhabitants). The municipalities with the critical intensity of occurrence are represented by the tone closest to dark red (very high) and red (high), in a moderate situation (Medium), by the tone closest to orange, and with the lowest intensity of occurrence in white (Low)(Figure 2).

## 4. Discussion

This study, when describing the profile of maternal mortality from COVID-19, showed a geographic distribution of cases in the health macro-region of Sobral, which can be attributed to the internalization process of the COVID-19 epidemic. (Track Changes).

Studies indicate that in order to understand the spread of COVID-19 in medium and small municipalities, one must consider the heterogeneity of indicators between health regions in terms of their structure of services and health surveillance, and social, cultural, and political aspects [12,13].

It was evidenced that in the period of 2020, the percentage was 5.8% of the number of deaths in pregnant and puerperal women due to COVID-19. These data reveal a downward trend in the number of deaths. On the other hand, studies carried out in states in the Northeast region point to an increasing trend in maternal mortality. Maternal mortality between 2007 and 2016 in Paraíba showed an increase of 61.5% [14,15].

Despite the mortality rates due to COVID-19 among pregnant and postpartum women in Brazil, they present a low percentage compared to other population groups, such as the elderly. A Brazilian study carried out between February and June 2020 recorded 978 pregnant and puerperal women diagnosed with COVID-19 and 124 maternal deaths in the country, a number 3.4 times greater than the total number of maternal deaths related to COVID-19 compared to the same period in the world [7]. However, Brazil presents a 2.4 times greater risk of adverse effects affecting more postpartum women than pregnant women [16].

In Brazil, efforts were made to implement strategies, such as the Rede Cegonha and the Ápice Project, for the qualification and improvement of maternal and child care in the country [17]. Another strategy adopted was the implementation of Maternal Mortality Committees, which aim to investigate maternal deaths, as well as analyze and point out preventive measures and are a way to support these implemented public policies [18].

In view of the individual characteristics of pregnant and postpartum women, it was noticed that most of the sample was in the 20 and 35 age group. It is noteworthy that the extremes of maternal age (≤15 or ≥35 years) are related to worse perinatal outcomes [19].

The findings are similar to research that described the clinical and epidemiological characteristics, risk factors, and outcome of COVID-19 in pregnant women in Aracaju, Sergipe, Brazil. The result showed that most pregnant women were aged between 20 and 34 years [20].

Corroborating with data from the Influenza Epidemiological Surveillance Information System (SIVEP-GRIPE), in which the age group most affected by COVID-19 in 2020 was 20 to 29 years old, with 2009 (41.2%) cases, followed by the age group of 30 to 39 years, with 1962 (40.2%) cases [21].

From the perspective of obstetrics, it is relevant to investigate race, this fact is justified by the fact that there are differences between pregnant women according to ethnicity. Dark-haired women have a greater genetic predisposition to diseases, especially hypertensive syndromes of pregnancy (SHG), and exposure to greater social vulnerability must be considered [22,23]. Therefore, the results pointed to the predominance of brown women.

The Birth in Brazil survey: National Survey on Childbirth and Childbirth, carried out in 2011/2012 with 23,894 women, revealed that black and brown women had the worst indicators of prenatal care and childbirth compared to white women. Black puerperal women had a higher risk of inadequate prenatal care, lack of connection to maternity, and absence of a companion. Racial inequalities were identified in the process of care during pregnancy and childbirth, showing a gradient from worse to better care among black, brown, and white women [24].

In this context, black women are more likely to have maternal mortality due to the coronavirus disease. This fact is also associated with the increase in the number of hospitalizations in more serious conditions, such as dyspnea and lower oxygen saturation, in addition to lower admissions to the Intensive Care Unit (ICU) and mechanical ventilation, with higher risks of death compared to white women [25,26].

It is known that racism prevented the adoption of preventive measures for COVID-19, considering that social distancing, the main measure proposed by the World Health Organization (WHO) at the beginning of the pandemic, was not a privilege for everyone. Brazil, where the black population represents the majority of informal workers, domestic service, commercial, and transport, was the hardest hit during the pandemic [27].

As for schooling, it was identified that pregnant and puerperal women had between 6 and 11 years of schooling, the data are similar to research carried out in Aracaju, in which maternal mortality occurs more in women with less than seven years of schooling. Therefore, the low level of education becomes a variable directly associated with a higher risk of maternal mortality, which may be due to the lack of knowledge of this population regarding the importance of prenatal care, routine monitoring of their newborn, and difficulty in accessing health equipment due to a lower social status and low education [28].

It is known that the recognition of the social determinants of health makes it possible to know the profile of pregnant and postpartum women, in order to reproduce horizontal and systematic health practices. Such information can help health professionals to develop healthcare models with an emphasis on problems that require attention and monitoring, such as maternal mortality from COVID-19 [29].

Regarding the epidemiological profile of pregnant and postpartum women due to COVID-19, it is identified that the majority were in the 3rd and 2nd trimesters of pregnancy. It is known that the main previous clinical conditions can be related to pregnancy complications in these periods, as well as worse perinatal outcomes, interfering with women’s health [30].

According to the results, it was noted that most pregnant and puerperal women had kidney disease as the most prevalent comorbidity. It is known that renal failure during pregnancy is a high maternal-fetal risk, leading to complications, such as hemorrhagic accidents, anemia, and risk of liver anomalies [31].

Research carried out in the state of Washington (USA), that analyzed 210 cases of hospitalized pregnant women diagnosed with COVID-19, showed that at least one comorbidity is linked, namely, asthma, hypertension, type 2 diabetes mellitus, autoimmune disease, and class obesity III. The result showed that patients with severe acute respiratory syndrome due to COVID-19 are more likely to develop severe forms of the disease [32].

A retrospective study in a national database in the United States showed the presence of gestational diabetes in patients with more critical conditions and who died [33].

Although COVID-19 mainly affects the lungs, other organs are affected in the course of the disease, especially the heart, liver, intestines, brain, testes, and kidneys. The reason why other organs are affected is due to the presence of ACE-2 in cells in the bloodstream [34,35].

It is noted that direct obstetric causes have shown a high incidence, which points to the need for health care that can prevent the death of this population. Studies revealed that direct obstetric causes were the main causes of death in pregnant women in the country [36,37].

The population of pregnant women has distinct characteristics regarding the presence of comorbidities, which requires quality care from health equipment [38]. It is perceived that adequate prenatal care is important, however, it was observed that the pandemic increased access barriers to prenatal care. These enclaves in the health system exacerbate the crisis in the health system [39].

A limitation of this study is the use of a secondary database with information regarding the studied population. He points out that, as the bank is continuously fed according to the last epidemiological week, there may not have been a lack of data.

## 5. Conclusions

The present study allowed us to describe the profile and distribution of maternal mortality from COVID-19 in the state of Ceará in the period 2020, highlighting the need to characterize pregnant and postpartum women, reinforcing the influence of social determinants of health as risk predictors for deaths from COVID-19. Thus, considering variables such as personal and obstetric history, sociodemographic and economic situation, contributes to resolute assistance in order to reduce risks during pregnancy. However, it points out the need for further studies on COVID-19 in pregnant/puerperal women due to the scarcity of information.

## Figures and Tables

**Table 1:** Care profile of COVID-19 cases in pregnant and postpartum women. Ceara, Brazil, 2020.

Disease Evolution	n	%
Confirmed cases	485	100.0
Confirmed cure	411	84.7
Confirmed deaths	28	5.8
Ward admission	463	95.5
ICU admission	61	12.6
Non-invasive ventilatory support	67	13.8
Invasive ventilatory support	35	7.2

**Source:** Rodrigues, A. and Lacerda, L. and Francisco, R.P.V. ‘Brazilian Obstetric Observatory’ arXiv preprint arXiv:2105.06534 (2021) [9].

**Table 2:** Characteristics of pregnant and postpartum women due to COVID-19 according to sociodemographic variables. Ceara, Brazil, 2020.

Profile	n (485)	%
Age group		
˂20	64	13
20–34	320	66
˃35	98	20
Race/skin color		
Asiatic	2	0.4
White	17	3.5
Aboriginal	1	0.2
Mixed	341	70.3
Black	2	0.4
Ignored	122	25.2
Place of residence		
Periurban	1	0.2
Rural	72	14.8
Urban	345	71.1
Ignored	67	13.8
Education		
No education	0	0.0
Middle school	21	4.3
Elementary school	29	6.0
High school	82	16.9
Graduation	18	3.7
Not informed	335	69.1

**Source:** Rodrigues, A. and Lacerda, L. and Francisco, R.P.V. ‘Brazilian Obstetric Observatory’ arXiv preprint arXiv:2105.06534 (2021) [9]. Note: Sample (n). Percentage (%). Trimester (tri). Elementary School.

**Table 3:** Epidemiological profile of COVID-19 cases in pregnant and postpartum women. Ceará. Brazil, 2020.

Variable	n (485)	%
Gestational age		
1st gestational trimester	26	5.4
2nd gestational trimester	45	9.3
3rd gestational trimester	320	66.0
Idade Gestacional ignorada	12	2.5
postpartum	82	16.9
Comorbidities		
Cardiovascular	21	4.3
Diabetes	18	3.7
Neuropathies	3	0.6
Pneumopatia	1	0.2
Kidney disease	67	13.8
Obesity	5	1.0

**Source:** Rodrigues, A. and Lacerda, L. and Francisco, R.P.V. ‘Brazilian Obstetric Observatory’ arXiv preprint arXiv:2105.06534 (2021) [9]. Note: Sample (n). Percentage (%). Trimester (tri). Elementary School.

## Data Availability

All data were taken from public the Brazilian Obstetric Observatory COVID-19 (OOBr COVID-19), which can be accessed through the link, https://observatorioobstetrico.shinyapps.io/covid_gesta_puerp_br/. Accessed on 8 November 2021.
